# A method for measuring spatial effects on socioeconomic inequalities using the concentration index

**DOI:** 10.1186/s12939-019-1080-5

**Published:** 2020-01-14

**Authors:** Sung Wook Kim, Hassan Haghparast-Bidgoli, Jolene Skordis-Worrall, Neha Batura, Stavros Petrou

**Affiliations:** 1 Division of Health Sciences, Warwick Medical School, University of Warwick, Coventry, CV4 7AL UK; 2 Institute for Global Health, University College London, London, UK; 3 Nuffield Department of Primary Care Health Sciences, University of Oxford, Oxford, UK

**Keywords:** Spatial analysis, Concentration index, HIV testing, Inequality, Malawi

## Abstract

**Background:**

Although spatial effects contribute to inequalities in health care service utilisation and other health outcomes in low and middle income countries, there have been no attempts to incorporate the impact of neighbourhood effects into equity analyses based on concentration indices. This study aimed to decompose and estimate the contribution of spatial effects on inequalities in uptake of HIV tests in Malawi.

**Methods:**

We developed a new method of reflecting spatial effects within the concentration index using a spatial weight matrix. Spatial autocorrelation is presented using a spatial lag model. We use data from the Malawi Demographic Health Survey (*n* = 24,562) to illustrate the new methodology. Need variables such as ‘Any STI last 12 month’, ‘Genital sore/ulcer’, ‘Genital discharge’ and non need variables such as Education, Literacy, Wealth, Marriage, and education were used in the concentration index. Using our modified concentration index that incorporates spatial effects, we estimate inequalities in uptake of HIV testing amongst both women and men living in Malawi in 2015–2016, controlling for need and non-need variables.

**Results:**

For women, inequalities due to need variables were estimated at − 0.001 and − 0.0009 (pro-poor) using the probit and new spatial probit estimators, respectively, whereas inequalities due to non-need variables were estimated at 0.01 and 0.0068 (pro-rich) using the probit and new spatial probit estimators. The results suggest that spatial effects increase estimated inequalities in HIV uptake amongst women. Horizontal inequity was almost identical (0.0103 vs 0.0102) after applying the spatial lag model. For men, inequalities due to need variables were estimated at − 0.0002 using both the probit and new spatial probit estimators; however, inequalities due to non-need variables were estimated at − 0.006 and − 0.0074 for the probit and new spatial probit models. Horizontal inequity was the same for both models (− 0.0057).

**Conclusion:**

Our findings suggest that men from lower socioeconomic groups are more likely to receive an HIV test after adjustment for spatial effects. This study develops a novel methodological approach that incorporates estimation of spatial effects into a common approach to equity analysis. We find that a significant component of inequalities in HIV uptake in Malawi driven by non-need factors can be explained by spatial effects. When the spatial model was applied, the inequality due to non need in Lilongwe for men and horizontal inequity in Salima for women changed the sign.

This approach can be used to explore inequalities in other contexts and settings to better understand the impact of spatial effects on health service use or other health outcomes, impacting on recommendations for service delivery.

## Background

Cultural factors contribute to the prevalence of the human immunodeficiency virus (HIV) in many sub–Saharan African settings [1]. In societies where multiple partners are socially allowed, the risk of transmitting HIV increases [2]. It is culturally permissible to have extra-marital affairs in many sub–Saharan African settings if a married couple cannot have babies, increasing the risk of the spread of the virus [3]. In cultural contexts where women are stigmatised when they receive an HIV test, early HIV detection to prevent mother to child HIV transmission (PMTCT) is challenging [4]. Relatively low education status is likely to contribute to lack of knowledge with respect to PMTCT, deterring women from receiving an HIV test.

Neighbourhood-wide factors contribute to uptake of HIV testing. Neighbourhood effects arise when the behaviour of a person is influenced by his/her neighbourhood mediated via its economy [5], politics [6] or health [7, 8], and underpin the measurement of geographical spillovers in the context of spatial analysis [9]. This concept has been broadly used in agricultural economics [10], ecological economics [11] and development economics [12]. This concept has also been used in research regarding the relationship between geography and crime rates [13] where the so-called “broken windows theory” [14] postulates that a crime rate in a certain area may increase if the area is a crime-ridden district as a result of interactions between those living in the area. Likewise, receipt of an HIV test may depend, in part, on neighbourhood effects. For example, in a society where HIV is tabooed, women living in the community may be less likely to receive an HIV test due to the risk of criticism of socially unacceptable behaviours. A study by Shacham and colleagues [15] conducted in the USA found that neighbourhood-level poverty is associated with poorer HIV management. Airhihenbuwa and colleagues [16] found through community-based research conducted in South Africa that AIDS-related stigma restricts attempts to receive voluntary counselling and testing and other HIV or acquired immune deficiency syndrome (AIDS) prevention efforts. The study by Kitara and colleagues [17] carried out by the Uganda AIDS Commission (UAC) found that only 36.1% of their research participants (youths in Gulu, Uganda) had undertaken an HIV test and the majority had not because of the fear of stigmatization. In short, there is compelling evidence that the uptake of HIV testing is associated with neighbourhood effects in many Sub-Saharan African settings.

As the coverage of antiretroviral therapy (ART) increases with the supports of global donors, such as the Global Fund, it is necessary to measure equity in access to and use of services to understand how the benefits of investment in HIV programmes are distributed across affected populations. This should be considered separately from increased coverage as argued by Moisi [18] who explained that “expansions in coverage do not always produce improvements in equity”.

Inequality in HIV testing in low- and middle-income countries, such as those in sub-Saharan Africa, is generally higher since inequality is strongly affected by socioeconomic factors such as gender or marital status [19, 20]. Nevertheless, there is paucity of evidence regarding equality in HIV testing in Malawi. No identified study of HIV testing uptake was carried out on a national sample, none studied equality and equity using the concentration index. We carried out a literature review using Pubmed with following keywords – Malawi, HIV test, socioeconomic status equality and equity [21]. After refining the search results several times based on the literature review [21], we obtained 7 relevant article [20, 22–27]. Among these, 3 articles were relevant about the association between socioeconomic status and HIV testing.

Obare et al. [20] studied the acceptance of population-based voluntary.

counselling and testing for HIV patients in rural Malawi. They found that women are more likely to be stigmatised when they want to get a HIV test than men.

Makwiza et al. [22] carried out the literature review of articles to examine equity on voluntary counselling and testing (VCT) in Malawi. They found that there is tendency that more women than men used HIV testing and counselling and an urban bias in provision of HIV testing and counselling. Conroy et al. [23] found women’s uptake on HIV test were more strongly affected by perceptions of a partner on HIV than their women’s own. As a result, our understanding of the potential barriers to achieving global goals in Malawi remains incomplete.

Socioeconomic inequality in health refers to the difference in health status, health service use or other health metric between socio-economic groups, with socio-economic status commonly measured using household wealth or income [28]. Approaches to analysing socioeconomic inequalities do not typically incorporate neighbourhood-wide factors or spatial effects in the health outcome of interest. At most, information on ‘location’ has been included in some equity analyses. For example, Kim and colleagues [29] used urban/rural location as a variable in their analysis of the determinants of socioeconomic inequality in HIV testing in Malawi. However, this does not capture the *spatial* effect of geographic location of HIV centres, for example, on HIV testing [22, 30, 31]. Jimenez-Rubio and colleagues [32] estimated the between-area concentration index in income-related inequalities in Canada using decomposition of the concentration index. The authors used the product of the population share and health variable share as a weight for each area, rather than using a spatial weight matrix [32–39]. However, previous studies have failed to capture the influence that spatial effects or spatial patterns, i.e. how the behaviour of a person is influenced by his/her neighbourhood, may have on the uptake of health services or on other health measures.

The number of HIV testing facilities and mobile programmes have increased in Malawi. For instance, in 2008, a provision of HIV counselling and testing to 500,000 pregnant female in Malawi was carried out at approximately 500 sites [21, 40]. It appears that a shift from facility-based testing to mobile testing to show a positive impact on access to HIV test, overcoming socioeconomic barriers to HIV test access.

Geographic location needs to be understood in the context of spatial effects. The geographic proximity of services to peoples’ homes is one of the significant factors affecting utilisation of health services, particularly in rural areas [41]. However, cultural obstacles may prohibit individuals from visiting facilities even if HIV testing clinics are located near the testee’s home [42]. As an example, Gwadz and colleagues [42] identified that lack of confidentiality surrounding doctors’ knowledge of who visits clinics to get tested can make the testee hide their presence at the HIV clinic. In addition, HIV prevalence tends to be higher in high-poverty neighbourhoods, compounding spatial patterns [43]. To sum up, a full understanding of the barriers to HIV testing needs to address spatial effects.

Assessments of spatial patterns in access to or use of HIV services are particularly important in high burden, low-income settings, such as Malawi. Firstly, HIV is an infectious disease showing spatial patterns of spread [43]. Secondly, HIV test centres are not spread equitably in geographic terms across Malawi [44, 45]. This may result in spatial disparities in HIV test rates across regions. Finally, as stigma remains a critical factor inhibiting access to HIV testing, women in rural areas, where people know each other well in addition to their family members, may feel inhibited from taking an HIV test [46, 47].

Two articles regarding spatial effect of socioeconomic status on health outcomes were found [48, 49]. However, these did not deal with the association between equity and health outcomes in the frame of spatial analysis. Understanding the spatial determinants of service access or use can therefore significantly expand our understanding of inequalities in health outcomes in resource-limited settings where individuals are more likely to suffer from multiple deprivations.

Thus, this study aims to estimate the contribution of spatial effects on inequality in uptake of HIV tests in Malawi, and develops a new methodological approach to the incorporation of spatial effects into equity analyses that can be applied in other health care contexts and settings.

## Methods

This section presents an overview of how the measurement of inequity using the concentration index can incorporate spatial effects.

### Concentration index

The standardized concentration index ( *C*_*h*_) is typically estimated using the following formula [50] The calculation of concentration index is explained in Additional file 1 Appendix 6:

1 $$ {C}_h=\frac{2 Cov\left({h}_i,{R}_i\right)}{\mu } $$

where *h*_*i*_ is health service use which in our case is taking HIV test, which takes value 1 if the individual i took the test or 0 otherwise for individual *i*, and the term *μ* is the mean of health service use, and R_*i*_ is individual i’s fractional socio-economic rank [29].

A nonlinear model of the relationship between a health variable and need and non-need variables can be expressed with a general functional form *G* [50]:

2 $$ \mathrm{y}=\mathrm{G}\left(\upalpha +{\sum}_{\mathrm{k}}{\upbeta}_{\mathrm{k}}{\mathrm{x}}_{\mathrm{k}}+{\sum}_j{r}_j{z}_j\right)+\upvarepsilon $$

where y is the health variable, *x*_*k*_ is a vector of need variables and *z*_*j*_ is a vector of non-need variables. K and j is the set of the need and non-need variables, respectively. α is a constant, *r*_*j*_ is a vector of coefficients of the non-need variables, and G can take the form of a probit, logit, Poisson or other estimator [50]. When outcome variables are probit, the increase in probability of a one-unit increase in a given variable associated with both of the values of the other independent variables and the initial value of the given variable [51].

To capture the spatial effect on health service use or health variable *y*, *G* uses a spatial lag model or spatial autoregressive model to estimate the concentration index [52]. In the spatial regression, a change in the explanatory variable for one region may not only affect its own region, but also the neighbouring regions, and in turn have other impacts on the original region. This impact can be captured with the coefficient of a spatial lag variable [9].

Non- need factors do an important role in estimating concentration index and health inequity. By including these factors, the result shows how access to HIV test is affected by socioeconomic status. Horizontal inequity is calculated by subtracting the contribution of need factors from the whole concentration index. As the concentration index is decomposed with need and non-need factors, it is essential to include non-need factors.

#### Spatial lag model

The spatial lag model can be used when the values of the dependent variable in one geographical unit are associated with the values of neighbouring geographical units [53]. The model is thus expressed as:

3 $$ y=\rho Wy+ X\beta +\upvarepsilon $$

where *y* is a vector of the dependent variable, *ρ* is a coefficient for a spatial lag variable, *W* is a spatial weight matrix ,*β* is a vector of regression coefficients and *e* is a vector of error terms [9]. This equation can be also presented in the following way.

3-1 $$ \left({I}_n-\rho W\right)y=+ X\beta +\upvarepsilon $$

3-2 $$ y={\left({I}_n-\rho W\right)}^{-1} X\beta +{\left({I}_n-\rho W\right)}^{-1}\upvarepsilon $$

*I*_*n*_ is a constant term vector and ρ is a scalar parameter. W corresponds to an n by n spatial weight matrix. Y is a n by 1 dependent variable vector and X is a dependent variables vector. Wy is a Spatial lag vector. and the scalar parameter ρ is the magnitude of spatial dependence [54]. As it can be seen from (3–2), the spatial effect is captured and presented with the parameters of each independent variable in the spatial lag model.

The spatial lag variable *y* is then expressed as follows:

4 $$ y=\left[\begin{array}{c}{y}_1\\ {}{y}_2\\ {}{y}_3\\ {}\vdots \\ {}{y}_n\end{array}\right]\kern0.5em \mathrm{Wy}=\left[\begin{array}{c}{\sum}_{j=1}^n{w}_{1j}{y}_j\\ {}{\sum}_{j=1}^n{w}_{2j}{y}_j\\ {}{\sum}_{j=1}^n{w}_{3j}{y}_j\\ {}\vdots \\ {}{\sum}_{j=1}^n{w}_{nj}{y}_j\end{array}\right]. $$

J is the first region in each row of the n x n spatial weight matrix W and n is the last region in each row of the matrix W. The spatial weight matrix is generated with an inverse distance weight matrix [9]. This is to capture the spatial effects of the uptake of HIV testing in our applied example. The weight matrix we used for a spatial lag model is an inverse distance weight matrix which is estimated with distance based neighbours rather than adjacency. There are two types of generating spatial weight matrix; one is making a weight based on distance and the other is making a weight based on contiguity. Adjacent points are often referred to as ‘first order’ points [55]. We did not consider neighbours of neighbours (second order contiguity). However, it is also possible to generate weight matrix using the concept of contiguity when DHS data is used. One study about measles vaccination coverage among children in Africa used queen contiguity for generating a spatial weight matrix [56].

Each component of the spatially lagged variable *Wy* presents the weighted average of the neighbouring regions of each index region *i*. W is the inverse distance weight matrix in this study [9]. This can be expressed as follows

5 $$ {W}_{ij}=\frac{1}{D\left(i,j\right)} $$

in which *D*(*i*, *j*) is the distance between places *i* and *j*. Row normalisation is performed to sum the weights to 1 in each row [9]. The spatial weight matrix was generated using ‘mata’ language in Stata (version 15), as the matrix generation process is computationally intensive given the large number of *n* x *n* matrices [57].

In general, equal treatment for equal need is referred to as horizontal equity [58]. ‘Equal access for equal need’ means that patients who have an equal need for a health service, make equal use of care without being disproportionately affected by nonneed factors such as socioeconomic status [58].

In estimating the concentration index, horizontal inequity (HI) is estimated by subtracting the contribution of need variables from the concentration index [29].

The relationship between a health variable and need and non-need variables is then as follows

6 $$ \mathrm{y}=\uprho Wy+{\sum}_{\mathrm{k}}{\upbeta}_{\mathrm{k}}{\mathrm{x}}_{\mathrm{k}}+{\sum}_j{r}_j{z}_j+\upvarepsilon $$

#### Socioeconomic inequality with spatial effects

Socioeconomic inequality can be estimated using a concentration index [28, 50]. The association between the health variable of interest (e.g. HIV test) and the rank of the socioeconomic status (e.g. education) determines the concentration index [50]. A change in the degree of income inequality, for example, does not affect the concentration index measure of income-related health inequality. In other words, regardless of the income inequality becomes higher (pro-rich) or lower (pro-poor) the inequality in income variables (or inequality in income distribution) of each observation in the data does not affect the concentration index in eq. (1).

Inequality due to socioeconomic factors is the sum of the product of the elasticity of non-need variables and the concentration index of the non-need variable [50]. It is presented in eq. (7):

7 $$ Inequality\  due\  to\ socioeconomic\ status\ (SES)=\sum \limits_{j=1}^j\frac{r_j{\overline{z}}_j{C}_j}{\mu } $$

where $\frac{r_j{\overline{z}}_j}{\mu }$ is the elasticity and ${\overline{z}}_j$ is the mean of the variable *j* and *μ* is the mean of the health variable of interest. Inequality due to need and non need factors is the sum of the contribution of need factors and non need factors to the whole CI.

Contribution is the degree of the contribution of need and non need variables to the whole concentration index.

This eq. (7) is also known as contributing to the decomposed concentration index [50]. *r*_*j*_ is the regression coefficient of the variable *j* if we estimate the non-linear *G* in eq. (2) using the probit model.

In the same manner, if we estimate *G* incorporating the spatial lag model, it is presented as follows:

8 $$ Inequality\  due\  to\  SES\  and\ spatial\ effect=\sum \limits_{j=1}^j\frac{{r^{\ast}}_j{\overline{z}}_j{C}_j}{\mu } $$

where *r*^∗^_*j*_ is the regression coefficient of a spatial probit model. The coefficients of the spatial lag model should be compared to the marginal effects of the probit model [54]. If a spatial effect does exist, eq. (7) will be different from eq. (8) indicating the inequality due to the spatial effect:

9 $$ Inequality\  due\  to\ spatial\ effect=\sum \limits_{j=1}^j\frac{r_j{\overline{z}}_j{C}_j}{\mu }-\sum \limits_{j=1}^j\frac{{r^{\ast}}_j{\overline{z}}_j{C}_j}{\mu } $$

Equation (9) presents the hidden spatial effect in the standard concentration index. If the inequality due to socioeconomic status (SES) in (7) is positive and the inequality due to SES and spatial effects is still positive in (8), but the magnitude of the difference between the first term and the second term in eq. (8) is smaller than the inequality due to SES in (7), i.e. (7) > (8), the result of eq. (9) will be positive but smaller than the result from eq. (8). If so, the positive sign of the result of eq. (9) means that pro-rich inequality is underestimated in eq. (8) due to the hidden spatial effect. In other words, the spatial effect actually contributes to the pro-rich inequality for the health variable of interest. Likewise, if both of eqs. (7) and (8) are negative and (9) remains negative (such that (7) < (8)), the absolute value of the result from eq. (8) is smaller than the result from (7). In this case, the hidden spatial effect contributes to pro-poor inequality for the health variable of interest. This association is summarised in Additional file 1 Appendix 1.

### Spatial autocorrelation

Spatial autocorrelation analysis can be applied to estimate the degree to which individuals with similar socioeconomic status live near to each other [59]. Spatial autocorrelation statistics depend on the definition of neighbourhood relations that can be expressed with Moran’s I index [60]*.* Consequently, we use Moran’s I index (eq. (10) to measure spatial autocorrelation, which ranges from – 1 to + 1:

10 $$ \mathrm{I}=\frac{N{\sum}_{i=1}^n{\sum}_{j=1}^n{W}_{ij}\left({X}_i-\overline{X}\right)\left({X}_j-\overline{X}\right)}{\sum_{i=1}^n{\sum}_{j=1}^n{W}_{ij}{\left({X}_j-\overline{X}\right)}^2} $$

Where *N* is the number of observations, $\overline{X}$ is the mean of the variable, *X*_*i*_ is the variable at location *i*, *X*_*j*_ is the variable at the location *j* and *W* is the spatial weight index. A negative value on Moran’s I indicates negative spatial autocorrelation while a positive value indicates positive spatial autocorrelation. Moran’s I larger than − 1/(N-1), which is the expectation of I under the null hypothesis, suggests that there is positive spatial autocorrelation for the variable of interest in the data [61].

If there exists no autocorrelation and then Moran’s I statistic is close to zero as the number of observations increases. A Moran’s I coefficient higher than −1/(*n* − 1) implies positive spatial autocorrelation, and a Moran’s I lower than −1/(*n* − 1) implies negative spatial autocorrelation.

### Data

This study uses data from the Malawian Demographic and Health Survey (DHS) collected in 2015–16 [62]. Data were collected from 11 districts in Malawi, namely Blantyre, Kasungu, Machinga, Mangochi, Mzimba, Salima, Thyolo, Zomba, Lilongwe, Mulanje and others [29]. The sample comprised women (*N* = 7289) and men (*N* = 17,273), 14–59 years of age. The dataset includes detailed information on knowledge of and attitudes related to HIV/AIDS, receipt of an HIV test, risky behaviours, HIV status and symptoms of sexually transmitted infections (STIs), in addition to socio-economic variables such as a wealth index and education level. The wealth index used converts the number or categories of assets available to individuals into quintiles [62]. *Education has 4 levels – no education, primary, secondary and higher education while literacy has 3 levels – cannot read at all, able to read only parts of sentence, able to read whole sentence.*

A separate education variable consists of four categories of highest education received: no education, primary education, secondary education and higher education. All individuals were given unique ID number and so there was not ‘double count’ in individuals.

The definition of the neighbourhood is determined at the cluster level due to data availability [63]. The location of DHS cluster in the data used in the spatial lag model is an estimated centre of the survey cluster [64]. In other words, DHS clusters are represented by point coordinates located at the centroid of each cluster with no adjustment made for different size clusters; also, these information were collected using Global Positioning System (GPS) receivers and validated by MEASURE DHS.

### Need and non-need variables

In general, equal treatment for equal need is referred to as horizontal equity [58]. ‘Equal access for equal need’ means that patients who have an equal need for a health service, make equal use of care without being disproportionately affected by nonneed factors such as socioeconomic status [58].

To estimate the concentration index in eqs. (2) and (6), need and non-need variables should be included [50]. STI symptoms are treated as need variables in this analysis, which is in line with other studies that have used symptoms as need variables in equity analyses [24, 29, 65]. The STI symptoms used in this study to reflect need are: non-ulcerative STIs (had any STD in last 12 months, had genital sore/ulcer in last 12 months, and had genital discharge in last 12 months) [29]. Education, literacy, wealth, marriage and gender are used as non-need variables in line with the existing literature [29] and included in eqs. (2) and (6).

The analysis was carried out using Stata 15 (College Station, TX, USA).

## Results

Table 1 presents reported HIV testing uptake for women and men in Malawi in 2015–16. Spatial level variables were presented in Additional file 1 Appendix 2. Results of decomposed index by district for women and men are presented in Additional file 1 Appendix 3 and 4.

**Table 1:** HIV testing by socio-economic status, Malawi DHS 2015–16

	Women	Tested (*N* = 5995)	*P*-value^b^	Men	*P*-value	
	Not tested (*N* = 1294)			Not tested (*N* = 2666)	Tested (*N* = 14,607)	
Region						
northern	181 (14)	1023 (17.1)		478 (17.9)	3121 (21.4)	
central	450 (34.8)	1770 (29.5)		1068 (40.1)	5129 (35.1)	
southern	663 (51.2)	3202 (53.4)	0.000	1120 (42)	6357 (43.5)	0.000
Education						
no	131 (10.1)	747 (12.5)		266 (10)	1635 (11.2)	
primary	846 (65.4)	3481 (58.1)		1740 (65.3)	8961 (61.3)	
secondary	300 (23.2)	1513 (25.2)		637 (23.9)	3611 (24.7)	
higher	17 (1.3)	254 (4.2)	0.000	23 (0.9)	400 (2.7)	0.000
Literacy						
Cannot read at al	320 (24.7)	1592 (26.6)		623 (23.4)	3874 (26.5)	
Able to read only part	144 (11.1)	461 (7.7)		256 (9.6)	1341 (9.2)	
Able to read whole sentence	830 (64.1)	3935 (65.6)	0.000	1787 (67)	9392 (64.3)	0.003
Wealth						
poorest	326 (25.2)	1497 (25.0)		342 (12.8)	2114 (14.5)	
poorer	274 (21.2)	1053 (17.6)		436 (16.4)	2666 (18.3)	
middle	248 (19.2)	1016 (16.9)		522 (19.6)	2722 (18.6)	
richer	212 (16.4)	1113 (18.6)		570 (21.4)	3002 (20.6)	
richest	234 (18.1)	1316 (22.0)	0.000	796 (29.9)	4103 (28.1)	0.011
Marriage						
No	1139 (88)	3775 (63.0)		1850 (69.4)	1846 (12.6)	
married	155 (12)	2220 (37.0)	0.000	816 (30.6)	12,761 (87.4)	
Any STI last 12 month^a^						
No	1284 (99.3)	5805 (97.0)		2629 (98.6)	14,202 (97.2)	
Yes	9 (0.7)	178 (3.0)	0.000	28 (1.1)	372 (2.5)	0.000
Genital sore/ulcer^a^						
No	1255 (97.1)	5436 (90.9)		2560 (96)	13,351 (91.4)	
Yes	38 (2.9)	545 (9.1)	0.000	100 (3.8)	1226 (8.4)	
Genital discharge^a^						
No	1265 (98)	5633 (94.1)		2592 (97.2)	13,753 (94.2)	
Yes	26 (2)	351 (5.9)	0.000	68 (2.6)	831 (5.7)	0.000

^a^: ‘don’t know’ was excluded

^b^: *P* value was estimated using Chi-2 test

There were significant differences in HIV testing uptake by socioeconomic factors. All socioeconomic factors of region, education, literacy, wealth and marriage differed significantly between those who were tested and those who were not tested amongst both women (*p* < 0.001) and men (*p* < 0.005). Amongst women who tested, the literate tended to take up HIV tests more (65.6%) than those who could not read (26.6%) or able to read only parts of sentences (7.7%). Women with primary education had a higher HIV test uptake rate (58.1%) than women with secondary (25.2%) or higher education (4.2%). The data also indicated that married women were less likely to take up HIV tests (37.0%) compared to single women (63.0%). Differences in uptake between the poorest and richest quintiles were small; HIV test uptake among the poorest female quintile was 25.0% compared to 22.0% among the richest female quintile.

Among men, the literate tended to take up HIV tests more (64.3%) than those who could not read (26.5%) or able to read only parts of sentences (9.2%). Similarly, men with a primary level of education had a higher HIV test uptake rate (61.3%) than men with secondary (24.7%) or higher education (2.7%). In contrast to women, married men were significantly more likely to take up a test (87.4%) than single men (12.6%; *P*-value< 0.001), whilst test uptake by men in the poorest quintile was significantly lower (14.5%; *P*-value = 0.011) than amongst men in the richest quintile (28.1%).

Table 2 describes the inequality due to need and non-need variables, their concentration indices and related contributions. We present the estimates for the probit and spatial probit estimators for the sub-sample of men in columns (A) and (B), respectively. Estimates for the probit and spatial probit estimators for the sub-sample of women are presented in columns (C) and (D), respectively.

**Table 2:** Results from the concentration index

			Men 2015–16		Women 2015–6	
			Probit (A)	Spatial probit (B)	Probit (C)	Spatial probit (D)
Need factors	Any STI last 12 month	Elasticity	−0.0017	− 0.0017	0.0104	0.0100
		CI	−0.0548	−0.0548	− 0.0477	− 0.0477
		Contribution	0.0001	0.0001	−0.0005	−0.0005
	Genital sore/ulcer	Elasticity	0.0014	0.0018	0.0418	0.0418
		CI	−0.0414	−0.0414	−0.0036	− 0.0036
		Contribution	−0.0001	−0.0001	− 0.0002	−0.0002
	Genital discharge	Elasticity	0.0030	0.0026	0.0054	0.0040
		CI	−0.0642	−0.0642	−0.0714	− 0.0714
		Contribution	−0.0002	−0.0002	− 0.0004	−0.0003
Non-need factors	Literacy	Elasticity	0.0098	0.0093	−0.0200	−0.0211
		CI	0.1133	0.1133	0.1208	0.1208
		Contribution	0.0011	0.0011	−0.0024	−0.0025
	Education	Elasticity	0.1001	0.0976	0.0438	0.0447
		CI	0.1352	0.1352	0.1514	0.1514
		Contribution	0.0135	0.0132	0.0066	0.0068
	Marriage	Elasticity	0.4680	0.4676	0.0652	0.0656
		CI	−0.0557	−0.0557	0.0014	0.0014
		Contribution	−0.0261	− 0.0260	0.0001	0.0001
	Wealth	Elasticity	0.0227	0.0184	0.0204	0.0087
		CI	0.2397	0.2397	0.2870	0.2870
		Contribution	0.0054	0.0044	0.0058	0.0025
	Inequality due to need		−0.0002	−0.0002	−0.0010	−0.0009
	Inequality due to non-need		−0.0060	−0.0074	0.0101	0.0068
	Horizontal inequity		−0.0057	−0.0057	0.0103	0.0102
	Goodness of fit (Pearson Chi2)		942.73		13,860.59	

Estimates from both models indicate that for men the inequality due to need is − 0.0002 (pro-poor). However, the inequality due to non-need variables was − 0.006 and − 0.0074 for the probit and spatial probit estimators, respectively. The horizontal inequity was the same for both models (− 0.0057). This result shows that men from lower socioeconomic groups are more likely to receive an HIV test after adjustment for spatial effects. This shows that spatial factors are associated with uptake of HIV testing, and part of the inequality due to non-need factors can be explained by spatial effects, indicating that the inequality estimated with the spatial lag model is in favor of lower socioeconomic groups (i.e., pro-poor).

For women, the inequality due to need was − 0.001 and − 0.0009 for the probit and spatial probit estimators, respectively. However, the inequality due to non-need variables was 0.01 and 0.0068 for the probit and spatial probit estimators, respectively. This suggests that the inequality was reduced when the spatial lag model was applied. The horizontal inequity was almost identical (0.0103 vs 0.0102) after applying the spatial lag model. This result shows that the captured spatial effects positively contribute to the reduced pro-poor inequality (from − 0.001 to − 0.0009) in receiving an HIV test.

Additional file 1 Appendix 3 and 4 present the concentration index and inequality results by need and non-need variables for each district in Malawi, separately for women and men. For women, the inequality due to socioeconomic factors including spatial effects tended to reduce the magnitude of inequality. The inequality due to non-need factors increased the pro-poor estimate in many districts such as Kasungu, Machinga, Mangochi, Mzimba, Salima and Thyolo. For example, the inequality due to non-need factors changed to 0.0261 from 0.027 in Kasungu and to − 0.007 from − 0.002 in Machinga. Inequality due to need factors did not change the sign when the spatial model was used. For women, inequality due to need factors did not change the sign when the spatial model was used. Horizontal inequity was highest in Kasungu (0.0606 and 0.0605) and lowest in Zomba (− 0.0162 and − 0.0156).

Similarly, for men, in the majority of districts the inequality due to non-need variables became more pro-poor when spatial effects were considered. Inequality due to need factors did not change the sign. Also in Blantyre, the inequality due to non need factors did not change at all (− 0.0027) whereas it has changed the sign in Lilongwe. horizontal inequity was highest in Lilongwe (0.0173 and 0.0168) while lowest in Salima (− 0.0313 and − 0.0310).

The results show that there are no consistent patterns in receipt of the HIV test. In most districts, the horizontal inequity did not change much and it was almost identical, regardless of incorporation of spatial factors. Also, the inequality due to non-need factors showed a tendency of becoming ‘pro-poor’ in most districts after applying the spatial model. However, one important point to note is that for men, the inequality coefficient due to non-need factors changed in sign in some districts. For example, the inequality due to non-need factors in Lilongwe changed significantly from positive (0.0029) to negative (− 0.0027) when the spatial lag model was used, indicating that incorporation of spatial effects generally reduce uptake of the HIV test. Given the higher degree of change in the contribution for ‘wealth’ variable (0.0227 to 0.0183) than the change of other variables, this may show the association between the spatial effect and the wealth variable. Similarly, for women in Salima, horizontal inequity has changed the sign when the spatial model was used.

**Fig. 1:**
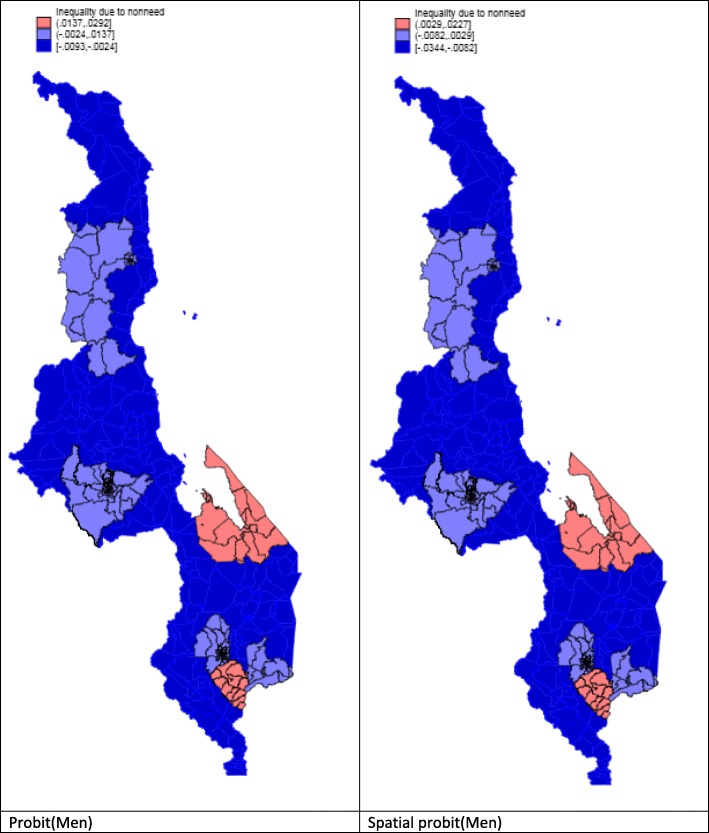
Inequalities by non-need factors for men

**Fig. 2:**
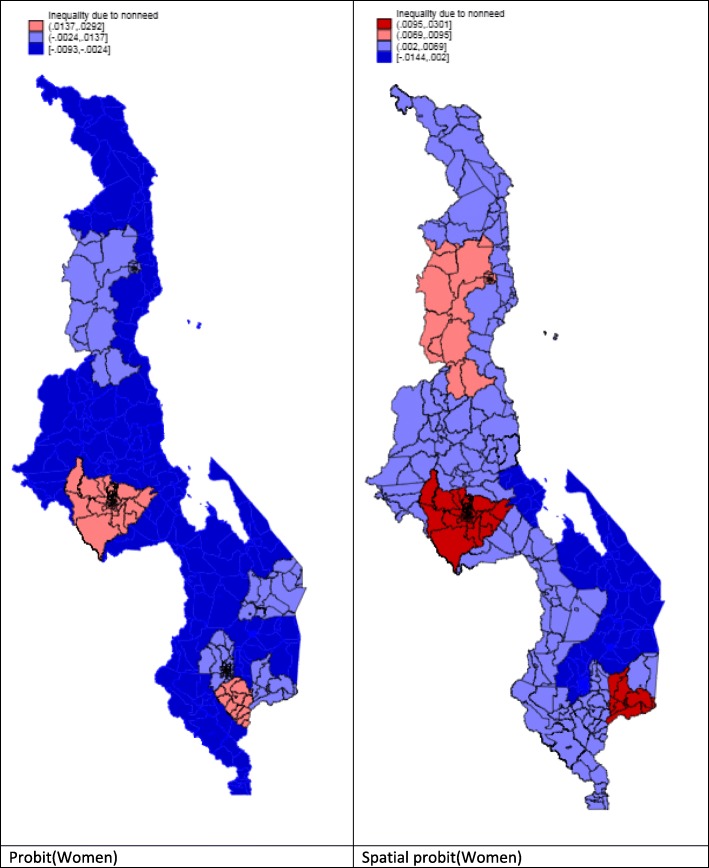
Inequalities by non-need factors for women

Figures 1 and 2 show the inequality due to socioeconomic variables such as education, literacy, wealth and marital status status, i.e. non-need variables, by districts. These figures show that women have a different pattern of inequality due to non-need variables when spatial regression is used. In contrast, men display an almost identical pattern of inequality, irrespective of whether or not spatial regression is used.

## Discussion

In this study, we introduce an approach to applying spatial analysis using a spatial weight matrix within the concentration index in order to improve our understanding of neighbourhood or spatial effects on health outcomes of interest. This study estimates inequalities in HIV test uptake associated with neighbourhood or spatial effects based on the spatial lag model. Using Malawian DHS data for 2015–16, we found that spatial effects on HIV test uptake are masked by other socioeconomic factors in the standard concentration index; that is, HIV test uptake becomes more pro-poor when spatial effects are captured by the new spatial lag model.

We introduced a new method of estimating the contribution of spatial effects on inequalities in health care utilisation. This method is not limited to HIV testing and can be applied to the use of other health services as well as other health outcomes. This method is most useful when it is expected that inequalities due to non-need factors are likely to be associated with a neighbourhood (spatial) effect. For the Malawian case study, we found that inequality due to non-need factors was generally reduced after incorporating spatial effects. Pro-rich inequality due to non-need factors are partly explained by spatial effects. This suggests that if HIV test uptake is determined by socioeconomic factors alone, it is likely to be pro-poor in Malawi based on the DHS dataset.

It was also found that horizontal inequity remained unchanged by the method introduced in this study. This is mainly because the contribution of need variables remained unchanged. Horizontal inequity can be measured by subtracting the need contribution from the unstandardized concentration index that is not affected by the spatial regression. The concentration index is determined only by the relationship between the variable of HIV testing and the rank of the living standards variable. In other words, the needs of individuals were not affected by neighbourhood or spatial effects. This is plausible as it is unrealistic to expect that the health needs of testees presenting with STI symptoms are directly affected by those of their neighbours. In brief, estimation of horizontal inequity in HIV testing in Malawi using the proposed approach is not particularly sensitive to neighbourhood or spatial effects.

In this study, we focus on the spatial lag model rather than the spatial error model. A spatial error model can be used when dependence in the regression error term is anticipated [52]. This is appropriate when the potential bias owing to the use of spatial data is expected. Mainly, the spatial error model can be considered due to the issue of omitted variables. However, our main purpose was to capture spatial interaction in the uptake of HIV testing among observations rather than correcting for biases that may potentially influence the spatial autocorrelation between the residuals of geographically close areas.

In the context of HIV test uptake in sub-Saharan African countries, our choice of model appears to be appropriate. Our main assumption in this study was that the uptake of HIV testing for individuals is associated with the uptake of HIV testing in their neighbours, in particular in the context of Sub-Saharan African settings. For example, we can consider a small town in which residents know each other well. In such a town, women cannot easily take up an HIV test due to taboos. Then, positive spatial autocorrelation will be observed in the town. As another example, we can assume that a new HIV testing facility is supplied in a certain area. Then, the rate of take up of HIV tests is likely to be higher in the area than other areas where there is no testing facility, regardless of whether we consider the culture of stigmatizing women receiving an HIV test. Given this reasoning, it was appropriate to use a spatial lag model in this study.

It may be argued that spatial effects reflect poverty effects and so there is no need to measure spatial factors separately from wealth. For example, evidence from studies conducted in the United States found that residential segregation is associated with urban poverty [66, 67]. Ethnic minorities disproportionately live in economically disadvantaged neighbourhoods. This, in turn, influences how residential areas shape health and contribute to racial disparities in health [68]. This suggests that there may be a correlation between spatial and poverty effects [69] and, thus, the analysis of one may capture the impact of the other. However, the relationship between spatial and poverty effects is neither complete nor causal. This is because the analysis of spatial effects can account for barriers to service uptake that differ from those that can be accounted for in analyses that only explore socio-economic effects, indicating the need to include spatial effects in more complex analyses and where policy relevant findings are required [70–72].

Based on the findings of this analysis, policy implications can be considered. Given the gender difference in uptake of HIV test, changing perceptions of female testing will be important. This can be carried out with campaigns such as the Malawi Radio Diaries programme in order to reduce gender inequality in HIV test uptake. Furthermore, considering the fact that the neighbourhood effect tends to make inequality due to non-need pro- rich, to change the male perception on HIV testing is critical as the changed perception will be captured in the neighbourhood effects as well.

A few limitations of this work should be noted. Firstly, Moran’s I was not significantly strong (0.0023 for women and 0.0019 for men) in this study (Additional file 1 Appendix 5). Accordingly, there is a possibility that the spatial effect was inappropriately represented in the regression model. However, as mentioned in the methods section, it is generally accepted that Moran’s I larger than the expectation of I under the null hypothesis indicate positive spatial autocorrelation and this condition was satisfied by our study. In addition, *P*-values for Moran’s I in this study were smaller than 0.001. As a result, it can be said that spatial autocorrelation exists in the use of HIV testing despite the weak magnitude. If we use a different dataset that captures stronger spatial autocorrelation and therefore has a bigger Moran’s I coefficient than that generated using the DHS 2015–16 Malawian data, the result of using the concentration index may vary and may reveal a greater contribution of non-need factors.

Secondly, the inverse distance weight matrix (and as a result the spatial lag variable) for the spatial regression was generated at a cluster level rather than an individual level. Multiple numbers of observations are given identical geographical information. Given this fact, there is a potential risk that this would not precisely represent the spatial interaction at an individual level. However, this is an inherent limitation in the DHS dataset. In practice, the definition of neighbourhoods is decided by available data. As aforementioned in the data section, it may be preferable to estimate neighbourhood effects at the ward level although it is unrealistic to expect that neighbourhoods follow ward boundaries.

Thirdly, we used a simplified interpretation on the coefficients of the spatial lag model. As mentioned earlier, we did not distinguish between direct and indirect effects within the spatial lag model. In the spatial lag model, a change in an independent variable may have effects both in the index region and neighbouring regions; in turn, this will affect the original region. This is the indirect effect of the spatial autocorrelation. We used the total effect that includes both direct and indirect effects; this is appropriate for our purpose of estimating the overall impact in each district.

Despite these limitations, the advantage of using our approach to estimating inequalities due to socioeconomic factors is that it is possible to capture spatial effects on the health variable of interest. In many cases, simply including a ‘rural-urban’ variable may not sufficiently estimate inequalities associated with socioeconomic status in developing countries since the variable is unlikely to fully capture spatial access to health facilities. Spatial access to health facilities is not only determined by urbanity but also by numerous factors, including male-dominating cultures, stigma, distance to the facilities and financial barriers. In fact, these factors are potential drivers to alter the estimates of non-need contributions in the concentration index; as such, our approach is expected to better estimate socio-economic inequalities in resource limited settings.

In conclusion, this study introduces a new methodological approach to incorporating spatial analysis into equity analysis. By doing so, we may better understand spatial and socioeconomic inequality in health service use in a range of settings where health services are not equally distributed in a geographic space. From the empirical analysis using Malawi DHS data, it was found that when the spatial model was applied, the inequality due to non need and horizontal inequity changed the sign in some districts. This implies the potential that the two districts of Lilongwe and Salima are sensitive to the spatial effect in HIV test uptake.

Further studies are needed to understand which spatial factors most significantly affect socioeconomic inequity in health service use across areas.

## Additional file

**Additional file 1.**

## Data Availability

Data can be obtained from the DHS website.

## Abbreviations

AIDS: Acquired Immune Deficiency Syndrome
ART: Antiretroviral Therapy
HIV: Human Immunodeficiency Virus
PMTCT: Prevent Mother to Child HIV Transmission
SES: Socioeconomic Status
