# Supplementary material for: A method for measuring spatial effects on socioeconomic inequalities using the concentration index
Source: Int J Equity Health. 2020 Jan 14;19:9. doi: 10.1186/s12939-019-1080-5 (PMC6958664; doi:10.1186/s12939-019-1080-5)
Supplement: Supplementary file 1 — Additional file 1. [file 12939_2019_1080_MOESM1_ESM.docx]

# Additional file 1

Appendix 1 : the association between the hidden spatial effect and sign.

|  | Equation (7) | (8) | Magnitude | (9) |
| --- | --- | --- | --- | --- |
| Sign | + | + | (7) > (8) | + |
|  | - | - | (7) < (8) | - |

**Appendix 2 : Spatial level variables used in the spatial model.**

| **Women** | **N** | **Mean** | **Standard deviation** | **Min** | **Max** | **Men** | **N** | **Mean** | **Standard deviation** | **Min** | **Max** |
| --- | --- | --- | --- | --- | --- | --- | --- | --- | --- | --- | --- |
| **Cluster** | 7,289 | 425.8 | 241.1 | 1 | 850 | **Cluster** | 17,273 | 425.1 | 245.6 | 1 | 850 |
| **Longitude** | 7,289 | 34.6 | 0.7 | 32.8 | 35.8 | **Longitude** | 17,273 | 34.5 | 0.7 | 32.8 | 35.8 |
| **Latitude** | 7,289 | -14.3 | 1.7 | -17.1 | -9.5 | **Latitude** | 17,273 | -14 | 1.8 | -17.1 | -9.5 |

**Appendix 3 : Results of decomposed index by district for women**

|  |  |  | **Lilongwe** |  | **Blantyre** |  | **Kasungu** |  | **Machinga** | | **Mangochi** | | **Mulanje** |  | **Mzimba** |  | | **Salima** |  | **Thyolo** |  | **Zomba** |  | **Other** |  |
| --- | --- | --- | --- | --- | --- | --- | --- | --- | --- | --- | --- | --- | --- | --- | --- | --- | --- | --- | --- | --- | --- | --- | --- | --- | --- |
|  |  |  | **Probit** | **Spatial** | **Probit** | **Spatial** | **Probit** | **Spatial** | **Probit** | **Spatial** | **Probit** | **Spatial** | **Probit** | **Spatial** | **Probit** | **Spatial** | | **Probit** | **Spatial** | **Probit** | **Spatial** | **Probit** | **Spatial** | **Probit** | **Spatial** |
| **Need** | **Any STI last 12 month** | **Elasticity** |  |  |  |  |  |  | 0.0355 | 0.0320 |  |  |  |  |  |  | |  |  | 0.0155 | 0.0167 | 0.0615 | 0.0611 | 0.0022 | 0.0018 |
|  |  | **CI** |  |  |  |  |  |  | -0.1804 | -0.1804 |  |  |  |  |  |  | |  |  | 0.0683 | 0.0683 | 0.0182 | 0.0182 | 0.0419 | 0.0419 |
|  |  | **Contribution** | |  |  |  |  |  | -0.0064 | -0.0058 |  |  |  |  |  |  | |  |  | 0.0011 | 0.0011 | 0.0011 | 0.0011 | 0.0001 | 0.0001 |
|  | **Genital sore/ulcer** | **Elasticity** | 0.0124 | 0.0125 | 0.0432 | 0.0433 | 0.0654 | 0.0648 | 0.1012 | 0.1044 | 0.0769 | 0.0754 | 0.0842 | 0.0845 |  |  | | -0.0132 | -0.0142 | 0.0812 | 0.0764 | 0.0767 | 0.0767 | 0.0535 | 0.0538 |
|  |  | **CI** | 0.0328 | 0.0328 | 0.0974 | 0.0974 | -0.3128 | -0.3128 | -0.0710 | -0.0710 | 0.0451 | 0.0451 | -0.0297 | -0.0297 |  |  | | 0.2789 | 0.2789 | 0.0572 | 0.0572 | 0.0618 | 0.0618 | -0.0429 | -0.0429 |
|  |  | **Contribution** | 0.0004 | 0.0004 | 0.0042 | 0.0042 | -0.0204 | -0.0203 | -0.0072 | -0.0074 | 0.0035 | 0.0034 | -0.0025 | -0.0025 |  |  | | -0.0037 | -0.0040 | 0.0046 | 0.0044 | 0.0047 | 0.0047 | -0.0023 | -0.0023 |
|  | **Genital discharge** | **Elasticity** | 0.0619 | 0.0631 |  |  | 0.0020 | 0.0024 |  |  | 0.0159 | 0.0143 | 0.0330 | 0.0336 | 0.0195 | 0.0212 | |  |  | -0.0180 | -0.0181 |  |  | 0.0039 | 0.0032 |
|  |  | **CI** | 0.1190 | 0.1190 |  |  | -0.1930 | -0.1930 |  |  | -0.1388 | -0.1388 | -0.1705 | -0.1705 | -0.0992 | -0.0992 | |  |  | -0.1860 | -0.1860 |  |  | -0.0953 | -0.0953 |
|  |  | **Contribution** | 0.0074 | 0.0075 |  |  | -0.0004 | -0.0005 |  |  | -0.0022 | -0.0020 | -0.0056 | -0.0057 | -0.0019 | -0.0021 | |  |  | 0.0033 | 0.0034 |  |  | -0.0004 | -0.0003 |
|  |  |  |  |  |  |  |  |  |  |  |  |  |  |  |  |  | |  |  |  |  |  |  |  |  |
| **Non-need** | **Literacy** | **Elasticity** | 0.0023 | 0.0021 | -0.0052 | -0.0077 | -0.0314 | -0.0318 | -0.0484 | -0.0520 | 0.0644 | 0.0590 | -0.0301 | -0.0294 | 0.0067 | 0.0069 | | -0.0263 | -0.0243 | -0.0281 | -0.0292 | -0.0849 | -0.0847 | -0.0255 | -0.0233 |
|  |  | **CI** | 0.1385 | 0.1385 | 0.0607 | 0.0607 | 0.0856 | 0.0856 | 0.1538 | 0.1538 | 0.1415 | 0.1415 | 0.0907 | 0.0907 | 0.1092 | 0.1092 | | 0.2220 | 0.2220 | 0.0693 | 0.0693 | 0.0989 | 0.0989 | 0.1139 | 0.1139 |
|  |  | **Contribution** | 0.0003 | 0.0003 | -0.0003 | -0.0005 | -0.0027 | -0.0027 | -0.0074 | -0.0080 | 0.0091 | 0.0083 | -0.0027 | -0.0027 | 0.0007 | 0.0008 | | -0.0058 | -0.0054 | -0.0019 | -0.0020 | -0.0084 | -0.0084 | -0.0029 | -0.0026 |
|  | **Education** | **Elasticity** | 0.1223 | 0.1226 | 0.0227 | 0.0241 | 0.0692 | 0.0633 | -0.0234 | -0.0198 | 0.0225 | 0.0126 | -0.0939 | -0.0936 | 0.1649 | 0.1627 | | -0.0164 | -0.0210 | 0.0079 | 0.0047 | 0.0270 | 0.0268 | 0.0483 | 0.0501 |
|  |  | **CI** | 0.1944 | 0.1944 | 0.1305 | 0.1305 | 0.1309 | 0.1309 | 0.1738 | 0.1738 | 0.1405 | 0.1405 | 0.1254 | 0.1254 | 0.1228 | 0.1228 | | 0.1855 | 0.1855 | 0.1106 | 0.1106 | 0.1479 | 0.1479 | 0.1407 | 0.1407 |
|  |  | **Contribution** | 0.0238 | 0.0238 | 0.0030 | 0.0031 | 0.0091 | 0.0083 | -0.0041 | -0.0034 | 0.0032 | 0.0018 | -0.0118 | -0.0117 | 0.0203 | 0.0200 | | -0.0030 | -0.0039 | 0.0009 | 0.0005 | 0.0040 | 0.0040 | 0.0068 | 0.0070 |
|  | **Marriage** | **Elasticity** | 0.0591 | 0.0598 | 0.0637 | 0.0634 | 0.0660 | 0.0665 | 0.0547 | 0.0556 | 0.1329 | 0.1310 | 0.0281 | 0.0282 | 0.0726 | 0.0756 | | 0.0741 | 0.0698 | 0.0524 | 0.0514 | 0.0536 | 0.0536 | 0.0673 | 0.0674 |
|  |  | **CI** | -0.0264 | -0.0264 | -0.0045 | -0.0045 | -0.0492 | -0.0492 | 0.1160 | 0.1160 | 0.0071 | 0.0071 | 0.0456 | 0.0456 | -0.0945 | -0.0945 | | 0.0320 | 0.0320 | -0.0372 | -0.0372 | -0.0062 | -0.0062 | 0.0055 | 0.0055 |
|  |  | **Contribution** | -0.0016 | -0.0016 | -0.0003 | -0.0003 | -0.0032 | -0.0033 | 0.0063 | 0.0065 | 0.0009 | 0.0009 | 0.0013 | 0.0013 | -0.0069 | -0.0071 | | 0.0024 | 0.0022 | -0.0020 | -0.0019 | -0.0003 | -0.0003 | 0.0004 | 0.0004 |
|  | **Wealth** | **Elasticity** | 0.0218 | 0.0247 | -0.0103 | -0.0018 | 0.0950 | 0.0947 | 0.0153 | 0.0132 | -0.0726 | -0.0819 | 0.0931 | 0.0944 | -0.0018 | -0.0187 | | 0.0068 | 0.0009 | 0.0311 | 0.0274 | -0.0105 | -0.0102 | 0.0199 | 0.0074 |
|  |  | **CI** | 0.3060 | 0.3060 | 0.1975 | 0.1975 | 0.2513 | 0.2513 | 0.3253 | 0.3253 | 0.3109 | 0.3109 | 0.2556 | 0.2556 | 0.2169 | 0.2169 | | 0.3168 | 0.3168 | 0.2479 | 0.2479 | 0.2660 | 0.2660 | 0.2893 | 0.2893 |
|  |  | **Contribution** | 0.0067 | 0.0076 | -0.0020 | -0.0004 | 0.0239 | 0.0238 | 0.0050 | 0.0043 | -0.0226 | -0.0255 | 0.0238 | 0.0241 | -0.0004 | -0.0041 | | 0.0022 | 0.0003 | 0.0077 | 0.0068 | -0.0028 | -0.0027 | 0.0058 | 0.0021 |
|  | **Inequality_need** | | 0.0078 | 0.0079 | 0.0042 | 0.0042 | -0.0208 | -0.0207 | -0.0136 | -0.0132 | 0.0009 | 0.0010 | -0.0081 | -0.0082 | -0.0019 | -0.0021 | | -0.0037 | -0.0040 | 0.0090 | 0.0089 | 0.0059 | 0.0059 | -0.0026 | -0.0025 |
|  | **Inequality_nonneed** | | 0.0292 | 0.0301 | 0.0003 | 0.0020 | 0.0270 | 0.0261 | -0.0002 | -0.0007 | -0.0093 | -0.0144 | 0.0106 | 0.0110 | 0.0137 | 0.0095 | | -0.0043 | -0.0068 | 0.0047 | 0.0034 | -0.0075 | -0.0074 | 0.0100 | 0.0069 |
|  | **Horizontal inequity** | | 0.0177 | 0.0176 | 0.0015 | 0.0015 | 0.0606 | 0.0605 | 0.0158 | 0.0154 | -0.0101 | -0.0102 | 0.0140 | 0.0141 | 0.0070 | 0.0071 | | -0.0001 | 0.0002 | -0.0060 | -0.0058 | -0.0163 | -0.0163 | 0.0113 | 0.0113 |
| Inequality due to need = contribution of need factors to the whole concentration index | | | | | | | | | | | | | | | | |  |  |  |  |  |  |  |  |  |
| Inequality due to non need Concentration index= contribution of non need factors to the whole concentration index | | | | | | | | | | | | | | | | |  |  |  |  |  |  |  |  |  |
| Horizontal inequity = whole CI - inequality due to need | | | | | | | | | | | | | | | | |  |  |  |  |  |  |  |  |  |

**Appendix 4 : Results of decomposed index by district for men**

|  |  | **Lilongwe** | | **Blantyre** | | **Kasungu** | | **Machinga** | | **Mangochi** | | **Mulanje** | | **Mzimba** | | | **Salima** | | **Thyolo** | | **Zomba** | | **Other** | |
| --- | --- | --- | --- | --- | --- | --- | --- | --- | --- | --- | --- | --- | --- | --- | --- | --- | --- | --- | --- | --- | --- | --- | --- | --- |
|  |  | **Probit** | **Spatial** | **Probit** | **Spatial** | **Probit** | **Spatial** | **Probit** | **Spatial** | **Probit** | **Spatial** | **Probit** | **Spatial** | **Probit** | **Spatial** | | **Probit** | **Spatial** | **Probit** | **Spatial** | **Probit** | **Spatial** | **Probit** | **Spatial** |
| **Need** |  |  |  |  |  |  |  |  |  |  |  |  |  |  |  | |  |  |  |  |  |  |  |  |
| **Any STI last 12 month** | **Elasticity** |  |  |  |  | -0.0053 | -0.0057 |  |  | 0.0617 | 0.0610 |  |  | -0.0111 | -0.0109 | | 0.0018 | 0.0009 | -0.0141 | -0.0140 | -0.0037 | -0.0012 | -0.0023 | -0.0023 |
|  | **CI** |  |  |  |  | 0.1308 | 0.1308 |  |  | -0.0060 | -0.0060 |  |  | 0.0012 | 0.0012 | | 0.3301 | 0.3301 | -0.1838 | -0.1838 | -0.1128 | -0.1128 | -0.0328 | -0.0328 |
|  | **Contribution** |  |  |  |  | -0.0007 | -0.0007 |  |  | -0.0004 | -0.0004 |  |  | 0.0000 | 0.0000 | | 0.0006 | 0.0003 | 0.0026 | 0.0026 | 0.0004 | 0.0001 | 0.0001 | 0.0001 |
| **Genital sore/ulcer** | **Elasticity** | 0.0784 | 0.0732 | 0.0103 | 0.0111 | 0.0189 | 0.0117 |  |  | 0.0030 | 0.0014 | 0.1375 | 0.1369 |  |  | | 0.0000 | 0.0001 | -0.0123 | -0.0177 |  |  | -0.0035 | -0.0028 |
|  | **CI** | -0.1082 | -0.1082 | -0.1308 | -0.1308 | 0.0166 | 0.0166 |  |  | -0.3257 | -0.3257 | -0.0968 | -0.0968 |  |  | | 0.0071 | 0.0071 | -0.0593 | -0.0593 |  |  | -0.0185 | -0.0185 |
|  | **Contribution** | -0.0085 | -0.0079 | -0.0013 | -0.0014 | 0.0003 | 0.0002 |  |  | -0.0010 | -0.0004 | -0.0133 | -0.0133 |  |  | | 0.0000 | 0.0000 | 0.0007 | 0.0011 |  |  | 0.0001 | 0.0001 |
| **Genital discharge** | **Elasticity** | -0.0037 | -0.0044 | 0.0511 | 0.0513 | 0.0499 | 0.0500 |  |  | -0.0124 | -0.0113 | 0.0529 | 0.0548 | -0.0029 | -0.0022 | | 0.0101 | 0.0099 | -0.0032 | -0.0038 | 0.1083 | 0.1145 | 0.0036 | 0.0031 |
|  | **CI** | 0.0753 | 0.0753 | -0.1910 | -0.1910 | 0.0248 | 0.0248 |  |  | 0.1237 | 0.1237 | -0.0753 | -0.0753 | -0.1413 | -0.1413 | | -0.1068 | -0.1068 | -0.0596 | -0.0596 | -0.0571 | -0.0571 | -0.0988 | -0.0988 |
|  | **Contribution** | -0.0003 | -0.0003 | -0.0098 | -0.0098 | 0.0012 | 0.0012 |  |  | -0.0015 | -0.0014 | -0.0040 | -0.0041 | 0.0004 | 0.0003 | | -0.0011 | -0.0011 | 0.0002 | 0.0002 | -0.0062 | -0.0065 | -0.0004 | -0.0003 |
| **Non-need** |  |  |  |  |  |  |  |  |  |  |  |  |  |  |  | |  |  |  |  |  |  |  |  |
| **Literacy** | **Elasticity** | 0.0055 | 0.0052 | 0.0223 | 0.0237 | 0.0316 | 0.0291 | 0.0336 | 0.0322 | -0.0290 | -0.0309 | -0.0197 | -0.0185 | 0.0396 | 0.0435 | | 0.0301 | 0.0319 | -0.0112 | -0.0121 | -0.0086 | -0.0080 | 0.0033 | 0.0031 |
|  | **CI** | 0.1194 | 0.1194 | 0.0649 | 0.0649 | 0.0747 | 0.0747 | 0.1881 | 0.1881 | 0.1226 | 0.1226 | 0.0946 | 0.0946 | 0.0795 | 0.0795 | | 0.1502 | 0.1502 | 0.1036 | 0.1036 | 0.1101 | 0.1101 | 0.1092 | 0.1092 |
|  | **Contribution** | 0.0007 | 0.0006 | 0.0014 | 0.0015 | 0.0024 | 0.0022 | 0.0063 | 0.0061 | -0.0036 | -0.0038 | -0.0019 | -0.0017 | 0.0031 | 0.0035 | | 0.0045 | 0.0048 | -0.0012 | -0.0013 | -0.0009 | -0.0009 | 0.0004 | 0.0003 |
| **Education** | **Elasticity** | 0.1152 | 0.1105 | 0.1054 | 0.1063 | 0.0923 | 0.0848 | 0.0197 | 0.0150 | 0.0575 | 0.0520 | -0.0124 | -0.0192 | 0.0488 | 0.0405 | | 0.0267 | 0.0282 | 0.0933 | 0.1006 | 0.0734 | 0.0708 | 0.1065 | 0.1046 |
|  | **CI** | 0.1679 | 0.1679 | 0.0947 | 0.0947 | 0.1151 | 0.1151 | 0.1572 | 0.1572 | 0.1378 | 0.1378 | 0.0788 | 0.0788 | 0.1089 | 0.1089 | | 0.1700 | 0.1700 | 0.1261 | 0.1261 | 0.1217 | 0.1217 | 0.1275 | 0.1275 |
|  | **Contribution** | 0.0193 | 0.0186 | 0.0100 | 0.0101 | 0.0106 | 0.0098 | 0.0031 | 0.0024 | 0.0079 | 0.0072 | -0.0010 | -0.0015 | 0.0053 | 0.0044 | | 0.0045 | 0.0048 | 0.0118 | 0.0127 | 0.0089 | 0.0086 | 0.0136 | 0.0133 |
| **Marriage** | **Elasticity** | 0.4939 | 0.4997 | 0.3520 | 0.3558 | 0.5545 | 0.5553 | 0.5521 | 0.5474 |  |  |  |  | 0.3613 | 0.3628 | | 0.4509 | 0.4521 | 0.2994 | 0.3121 | 0.4886 | 0.4895 | 0.4783 | 0.4780 |
|  | **CI** | -0.0804 | -0.0804 | -0.0401 | -0.0401 | -0.0425 | -0.0425 | -0.0590 | -0.0590 |  |  |  |  | -0.0591 | -0.0591 | | -0.0694 | -0.0694 | -0.0374 | -0.0374 | -0.0707 | -0.0707 | -0.0540 | -0.0540 |
|  | **Contribution** | -0.0397 | -0.0402 | -0.0141 | -0.0143 | -0.0236 | -0.0236 | -0.0326 | -0.0323 |  |  |  |  | -0.0214 | -0.0214 | | -0.0313 | -0.0314 | -0.0112 | -0.0117 | -0.0345 | -0.0346 | -0.0258 | -0.0258 |
| **Wealth** | **Elasticity** | 0.1045 | 0.0842 | 0.0002 | 0.0000 | 0.0800 | 0.0611 | -0.0088 | -0.0310 | 0.0740 | 0.0349 | -0.0031 | -0.0161 | 0.0455 | 0.0236 | | -0.0438 | -0.0363 | 0.0149 | 0.0428 | -0.0073 | -0.0107 | 0.0155 | 0.0127 |
|  | **CI** | 0.2168 | 0.2168 | 0.1300 | 0.1300 | 0.2526 | 0.2526 | 0.2778 | 0.2778 | 0.2473 | 0.2473 | 0.2153 | 0.2153 | 0.2226 | 0.2226 | | 0.2789 | 0.2789 | 0.2439 | 0.2439 | 0.2347 | 0.2347 | 0.2403 | 0.2403 |
|  | **Contribution** | 0.0227 | 0.0183 | 0.0000 | 0.0000 | 0.0202 | 0.0154 | -0.0024 | -0.0086 | 0.0183 | 0.0086 | -0.0007 | -0.0035 | 0.0101 | 0.0053 | | -0.0122 | -0.0101 | 0.0036 | 0.0104 | -0.0017 | -0.0025 | 0.0037 | 0.0030 |
| **Inequality_need** |  | -0.0088 | -0.0083 | -0.0111 | -0.0112 | 0.0009 | 0.0007 |  | 0.0000 | -0.0029 | -0.0022 | -0.0173 | -0.0174 | 0.0004 | 0.0003 | | -0.0005 | -0.0008 | 0.0035 | 0.0039 | -0.0058 | -0.0064 | -0.0002 | -0.0002 |
| **Inequality_nonneed** | | 0.0029 | -0.0027 | -0.0027 | -0.0027 | 0.0096 | 0.0038 | -0.0256 | -0.0325 | 0.0227 | 0.0120 | -0.0035 | -0.0067 | -0.0028 | -0.0083 | | -0.0344 | -0.0319 | 0.0030 | 0.0102 | -0.0283 | -0.0294 | -0.0082 | -0.0091 |
| **Horizontal inequity** | | 0.0173 | 0.0168 | -0.0058 | -0.0056 | 0.0067 | 0.0068 | -0.0253 | -0.0253 | 0.0256 | 0.0250 | 0.0152 | 0.0153 | -0.0003 | -0.0002 | | -0.0313 | -0.0310 | 0.0033 | 0.0029 | -0.0162 | -0.0156 | -0.0083 | -0.0083 |
| Inequality due to need = contribution of need factors to the whole concentration index | | | | | | | | | | | | | | | |  |  |  |  |  |  |  |  |  |
| Inequality due to non need Concentration index= contribution of non need factors to the whole concentration index | | | | | | | | | | | | | | | |  |  |  |  |  |  |  |  |  |
| Horizontal inequity = whole CI - inequality due to need | | | | | | | | | | | | | | | |  |  |  |  |  |  |  |  |  |

**Appendix 5 : Moran’s I**

|  | **Simulation** | **Moran's I** | **Standard deviation** | **-1/(n-1)** |
| --- | --- | --- | --- | --- |
| **Men** | 1,000 | 0.0019 | 0.0059 | -0.0001 |
| **Women** | 1,000 | 0.0023 | 0.0050 | -0.0001 |

**Appendix 6 :** Calculation of the concentration index

A standard concentration index (CI) is calculated as

|  | ${CI}_{y}=\frac{2Cov\left( y_{i},R_{i} \right)}{\mu}$ | (1) |
| --- | --- | --- |

where is CI_y_ is the concentration index for health service use y. it can be calculated by estimating covariance between y and fractional socio-economic rank of individual. $y_{i}$ is health service use for individual i, $\mu$ is the mean of health service use, and $R_{i}$ is individual i’s fractional socio-economic rank. The value of this indicator ranges -1 to 1. CI_y_ shows a positive sign if the health service use is pro- rich in terms of $R_{i}$; but shows a negative sign if the health service use is pro-poor in terms of $R_{i}$.

It can be assumed that health service use is decided by a set of k independent variables ($x_{k}$)

|  | $y=\alpha+\sum_{k} \beta_{k}x_{k}+\varepsilon$ | (2) |
| --- | --- | --- |

where $\beta_{k}$ is a set of coefficients and $\varepsilon$ is the error term. The concentration index for y (health service use) can be presented as

|  | $CI=\sum_{k} \frac{\beta_{k}\bar{x}_{k}}{\mu}C_{k}+e/\mu$ | (3) |
| --- | --- | --- |

where $C_{k}$ is the concentration index of $x_{k}$ and $\bar{x}_{k}$ is the mean of the independent variable$x_{k}$. $e$ is the generalized concentration index for the error term. This error term is the remaining unexplained inequality in the model [16].
